# Impact of Microcystin-LR on Liver Function Varies by Dose and Sex in Mice

**DOI:** 10.3390/toxins10110435

**Published:** 2018-10-28

**Authors:** Igor Mrdjen, Mark A. Morse, Randall J. Ruch, Thomas J. Knobloch, Shambhunath Choudhary, Christopher M. Weghorst, Jiyoung Lee

**Affiliations:** 1 College of Public Health, Division of Environmental Health Sciences, The Ohio State University, Columbus, OH 43210, USA; igormrdjen10@gmail.com (I.M.); knobloch.1@osu.edu (T.J.K.); weghorst.2@osu.edu (C.M.W.); 2 Charles River Laboratories, Spencerville, OH 45887, USA; Mark.Morse@crl.com (M.A.M.); Shambhunath.Choudhary@crl.com (S.C.); 3 Department of Cancer Biology, University of Toledo, Toledo, OH 43614, USA; Randall.Ruch@utoledo.edu; 4 Department of Food Science & Technology, The Ohio State University, Columbus, OH 43210, USA

**Keywords:** liver health, ingestion, acute exposure, harmful algal blooms, female and male, cyanotoxin

## Abstract

Microcystin (MC) exposure is an increasing concern because more geographical locations are covered with cyanobacterial blooms as eutrophication and bloom-favoring environmental factors become more prevalent worldwide. Acute MC exposure has been linked to gastrointestinal distress, liver toxicity, and death in extreme circumstances. The goal of this study was to provide an accurate and comprehensive description of MC-LRs impacts on liver pathology, clinical chemistry, and gap junction intercellular communication (GJIC) in CD-1 male and female mice. Mice were exposed to 0, 3000, and 5000/4000 µg/kg/day MC-LR, daily for 7 days, and were necropsied on Day 8. Blood samples for clinical chemistry analysis were processed to serum, while liver sections were fixed for histopathology or evaluated for GJIC using fluorescent cut-load dye. Results show a dose-dependent relationship with MC-LR exposure and hepatocellular hypertrophy, degradation, and necrosis. Clinical chemistry parameters alanine aminotransferase, aspartate aminotransferase, alkaline phosphatase, total bilirubin, and cholesterol increased significantly in MC-LR exposed mice. Clinical chemistry parameter analysis showed significantly increased susceptibility to MC-LR in females compared to males. Changes in GJIC were not noted, but localization of hepatotoxicity near the central veins and midlobular areas was seen. Future toxicity studies involving MCs should consider response differences across sexes, differing MC congeners, and combinatorial exposures involving other cyanotoxins.

## 1. Introduction

Exposure to cyanotoxins, specifically microcystins (MCs), has been shown to produce various adverse health outcomes in humans and animals. Direct contact with high concentrations of MCs can cause rashes as well as skin and eye irritation [1]. Ingestion of MCs has been shown to result in nausea and gastrointestinal distress [1,2]. Extreme cases of MC ingestion can cause animal and human liver toxicity and even death [3,4,5]. While proper treatment methods can curb exposure risks to MC, economically stressed areas still do not have the ways to intervene to address MC exposures, and early warning based on real-time monitoring systems are absent [6,7,8,9,10].

Therefore, it is imperative to understand the health outcomes resulting from mammalian MC ingestion. This study focuses on liver health outcomes associated with the ingestion of microcystin-LR (MC-LR), the most common and toxic congener of MC [11]. Ingestion of MC-LR has been widely studied, and has shown several trends in preclinical animal models. While several animal models of exposure have been employed, mice and rats are the most commonly utilized mammalian models [11,12,13]. These studies consistently note increased sensitivity of mouse models compared to rats while controlling for dose and body weight differences [14]. Further, variations in MC exposure times and time to sacrifice vary across study designs making specific outcomes difficult to compare across studies. Experimental designs have varied in duration ranging from immediate impacts of MC-LR ingestion (30 min. post-exposure) [15], to sub-chronic impacts of exposure (up to a 21-day treatment period) [16]. Dosage and frequency of exposure vary widely across study designs, ranging from a single dose of MC-LR to daily exposures for extended periods of time [17,18].

Along with variations in study design and animal model use, the existing knowledge from published preclinical studies describes varied outcomes related to the acute exposure to MC. Studies have reported changes in an assortment of clinical chemistry parameters, including alterations in the “aspartate aminotransferase (AST) to alanine aminotransferase (ALT)” ratio and levels of glutathione-S-transferase (GST) [19,20]. AST/ALT ratios are often used as markers of liver damage, with outcomes such as non-alcoholic liver disease producing 4-fold increases in AST/ALT ratio [21]. Other studies, however, report decreases in AST levels in mice, leading to a lower AST/ALT ratio [22]. Consequently, the applicable value of this biomarker ratio remains unclear. Numerous studies have also shown changes in GST concentrations in serum during MC exposure. GST is a Phase II enzyme intrinsic to the detoxification of MC-LR [23]. Fluctuations of GST enzyme levels and activity in serum are expected throughout cyanotoxin exposure as GST is depleted during MC-LR detoxification in the liver [19,24].

Pathological analysis during toxicological studies have shown mortality and liver toxicity attributed to hemorrhaging, hepatocellular necrosis, and the presence of pro-inflammatory responses [17,25]. The transport of MCs into the liver and uptake into the hepatocyte by the organic anion-transporting polypeptide 1B2 make the liver the primary target organ of MC-LR toxicity [26]. Clinical and pathological outcomes can be attributed to the covalent binding of MC compounds to the catalytic subunits on protein phosphatases 1 (PP1) and 2A (PP2A) [27]. This binding results in inhibition of PP1/2A leading to hyperphosphorylation of cellular microtubules, loss of cellular structure, and production of oxidative stress leading to cellular apoptosis [28].

Relative toxicities and lethal doses of MC-LR vary across exposure methods and animal models. MC exposure protocols frequently use intraperitoneal (i.p.) injection or oral gavage administration in order to control for measured dosing and delivery [12,25,29,30,31]. However, these two standard practice exposures for MC-LR toxicity produce drastically different lethal dose (LD50) estimates, with i.p. injection often producing a 10-fold higher toxicity compared to oral gavage [14]. The large variation in adverse health outcomes demonstrates the necessity for a more complete understanding of MC-LR toxicity, and shows a continuing gap in measured wellness metrics, while also providing evidence of histopathological and biochemical alterations with near-lethal and sub-lethal exposures to MC-LR.

Finally, while many studies have focused on clinical chemistry and pathological outcomes of MC-LR exposure, few have investigated the impacts of MC-LR on cellular communication and signaling. Signaling functions and transport of sugars, water, and small molecules in all vertebrate cells are performed through gap junction intercellular communication (GJIC) [32]. Alterations or disruptions in GJIC have been linked to diabetes, autoimmune disorders, cancers, and neuropathy [33]. GJIC transport molecules can also facilitate the transport of certain toxins across cells, modifying the toxicity of certain compounds [34]. Studies have demonstrated that interaction with non-genotoxic agents also inhibit GJIC [35,36]. Since MC-LR has been associated with increased cancer incidence, yet has been shown to be non-genotoxic, it is expected that GJIC would be inhibited in vivo following exposure to MC-LR [37,38].

The goal of this study was to accurately measure liver health outcomes following acute MC-LR exposure via ingestion in mice. We investigated the impacts of MC-LR ingestion on the pathology, clinical chemistry parameters, and GJIC in both male and female CD-1 mice in. The focus of these studies was to understand the differences in health outcomes at defined MC-LR doses representing near-lethal and sub-lethal concentrations. We targeted our assessment to accurately examine the dose-response relationship between MC-LR and validated histopathological, clinical chemistry, and GJIC outcomes measurements.

## 2. Results

### 2.1. Mouse Mortality, Health, and Clinical Observations

During Phase A, no MC-LR related mortalities, nor clinical changes, were recorded. All males, aside from one male (group 3), experienced slight net body weight losses during the study. Female groups 1 and 2 gained weight within normal limits, while group 3 females gained only 0.15 g. Based on Charles River Labs historical data, male and female CD-1 mice of this age are expected to gain ~1.5 g and ~1.0 g body weight per week, respectively.

In Phase B, multiple mortalities related to MC-LR were noted in female group 3 (5000/4000 µg/kg/day). The first mortality occurred less than 3 hours following the only injection of 5000 µg/kg/day, with the second coming prior to dosing on Day 2, in the same group. Consequently, MC-LR dosage of group 3 was lowered from 5000 to 4000 µg/kg/day, beginning on Day 2. On Day 7, a third group 3 female was euthanized moribund with clinical signs including: Abnormal gait, decreased activity, apparent hypothermia, labored breathing, and decreased fecal output. Only one male mortality was noted in Phase B, occurring on Day 4 in Group 2 (3000 µg/kg/day). Histopathological analysis of deceased mice found signs of marked perisinusoidal hemorrhaging and liver necrosis. Examination of the euthanized moribund female showed moderate necrosis and degeneration in the liver.

Apart from the clinical signs associated with the moribund euthanasia of the group 3 female, clinical signs were only found on Day 8. Clinical signs on Day 8 consisted of decreased fecal output in one group 2 female, and hunched posture and decreased fecal output another group 2 female. These signs were regarded as MC-LR related. 

Group 3 males showed a decrease in body weight gain compared to group 1, resulting in ~7% lower body weight than controls. Four group 3 females showed a decrease in body weight (0.3 to 3.8 g of body weight lost from Day 1 to 8). Females in group 2 had lower mean body weight gain than group 1 throughout the study, leading to a ~5% lower body weight that group 1. The mentioned changes in body weight were minimal and not dose related. No clear MC-LR effects on body weight gain in group 2 males or group 3 females were seen.

### 2.2. Clinical Chemistry

**Figure 1:**
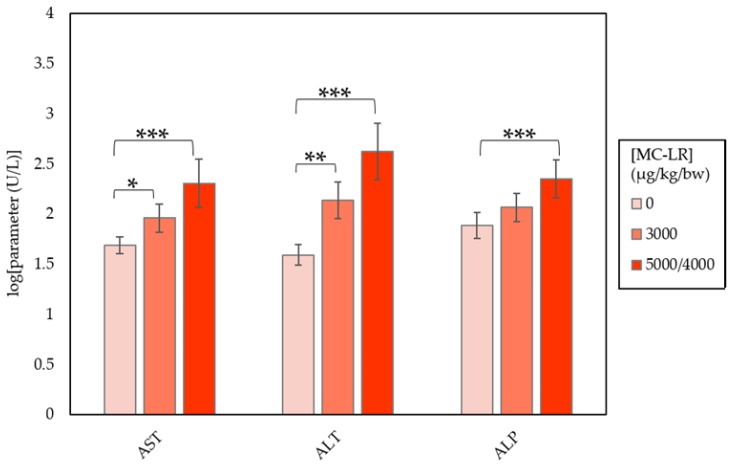
CD-1 male mouse clinical chemistry parameters by exposure group. Groups were compared to the 0 µg/kg/bw group using Dunn’s test. Differences in results were significant at a levels of 0.05 (*), 0.01 (**), and 0.001 (***).

**Figure 2:**
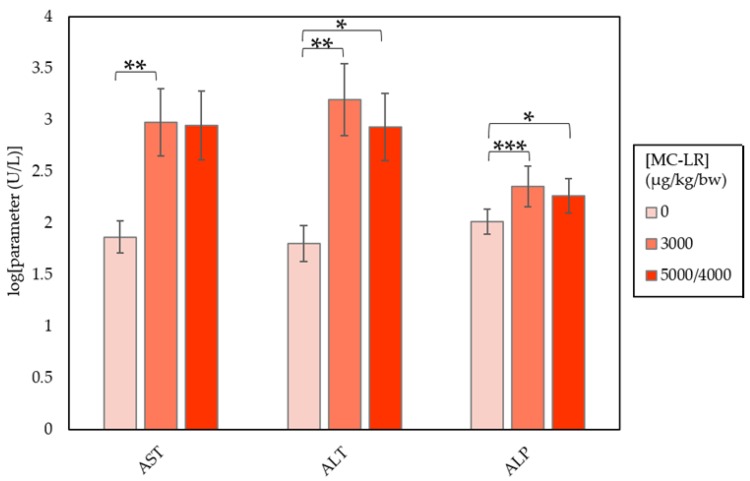
CD-1 female mouse clinical chemistry parameters by exposure group. Groups were compared to the 0 µg/kg/bw group using Dunn’s and Dunnett’s tests. Differences found via Dunn’s test were significant at *a* levels of 0.05 (*), 0.01(**), and 0.001(***).

Clear alterations in clinical chemistry parameters were seen in males and females. Males (group 2) and females (group 2) administered 3000 µg/kg/day MC-LR displayed, at varying degrees of significance, elevations of AST, ALT, ALP, and bilirubin (Table 1 and Table 2). Elevations in cholesterol levels were also seen in group 2 females, while blood glucose levels declined 0.8-times (*p* ≤ 0.001) and 0.7-times (*p* ≤ 0.01) in groups 2 males and females, respectively. Changes in clinical chemistry were generally dose related in males (Figure 1), but not in females (Figure 2). Additionally, variations in clinical chemistry across individual mice were seen, showing elevations in bilirubin and/or serum enzymes beyond the previously mentioned parameters. Overall, female mice experienced higher magnitudes of clinical chemistry change than males (Table 3).

### 2.3. Histopathology

During Phase A, minimal hepatocellular hypertrophy was noted in exposed male mice, but not in female mice. Minimal to mild hepatocellular hypertrophy occurred in a dose-dependent manner in males and females. Hypertrophy was characterized by enlarged hepatocytes around central veins and midlobular areas, and was more pronounced in males than females (Table 4).

At the conclusion of Phase B, minimal to mild hepatocellular degeneration was seen in both sexes. Hepatocellular degeneration was characterized by the presence of swollen and individualized hepatocytes with pale nuclei and vacuolated cytoplasm. Hepatocellular necrosis ranged from minimal to moderate in surviving mice across sexes, and was characterized by hepatocytes exhibiting hypereosinophilic cytoplasm, nuclear pyknosis, karyorhexis, and karyolysis. Generally, these changes were dose-dependent and localized near central veins and midlobular areas, with degeneration progressing to necrosis. In severely affected livers, massive necrosis was characterized by dissociation of hepatic cords, with abundant hemorrhage, necrotic debris, degenerate neutrophils, and Kupffer cells in place (Table 4). Mild hemorrhaging, characterized by presence of extravasated erythrocytes around the sinusoids and within the liver parenchyma, was seen in the group 3 males (Table 4). Early death animals generally had moderate to marked hepatocellular necrosis and marked hemorrhage.

### 2.4. Gap Junction Intercellular Communication

**Figure 3:**
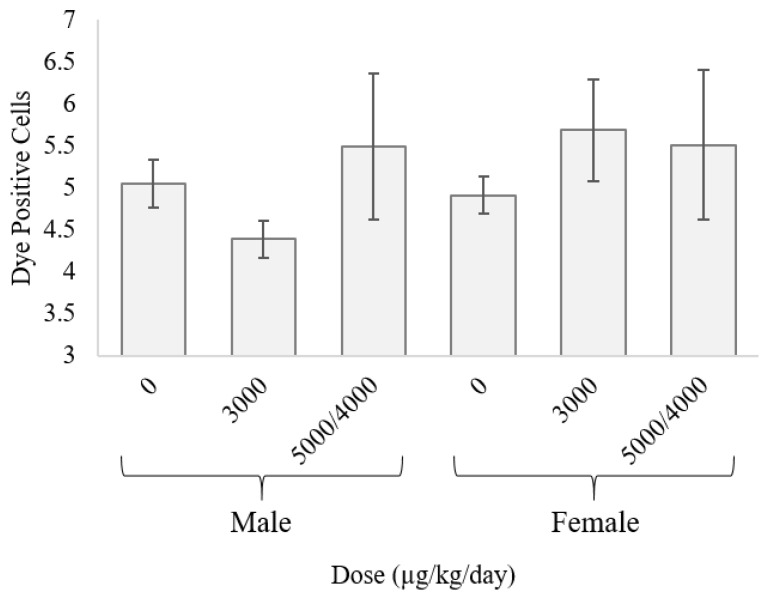
Hepatocellular gap junction communication as measured by mean dye-positive cell count. No significant differences in gap junction communication were seen across treatments or sexes.

Microscopic examination of fluorescent dye cut-loaded hepatic tissue showed no statistically significant dose-dependent changes in GJIC across sexes (Figure 3).

## 3. Discussion

The goal of this study was to accurately describe the pathological, clinical chemistry, and GJIC related outcomes attributed to MC-LR ingestion in mice across sex and exposure dose. Phase A of the study aimed to determine a tolerable, sub-lethal MC-LR dose. Upon exposure at varying intervals, data and observations of two male and two female mice were used to determine whether exposure to concentrations near 2000 µg/kg/day would result in excessive mortality. Hepatocellular hypertrophy was observed in both daily-exposed (Group 3) male mice in Phase A, but not the female mice. Based on findings from Phase A and existing literature, doses of 3000 µg/kg/day and 5000 µg/kg/day were selected to represent sub-lethal and near-LD50 MC-LR exposures [1,12,14,25,29,30]. While the LD50 values cited in past studies range from 3000–10,000 µg/kg/day varying by mouse strain, sex, and exposure duration, we found the LD50 for orally administered MC-LR in CD-1 mice to be greater than 5000 µg/kg/day [1,14,17,39]. However, due to two observed mortality events in Group 3 females (5000 µg/kg/day MC-LR), the MC-LR dose was adjusted to 4000 µg/kg/day on the second day of dosing.

Upon necropsy on Day 8, histopathologic examination of collected liver tissue showed that premature death in 3 of 4 mice was attributed to marked intrahepatic hemorrhaging related to MC-LR exposure [26]. Hepatocellular hypertrophy, similar to Phase A, was observed in Group 2 males (80%) and females (20%) exposed to 3000 µg/kg/day (Table 4). Mice exposed to 5000/4000 µg/kg/day demonstrated higher rates of hypertrophy, with Group 3 males and females showing 100% and 90% incidence of hypertrophy, respectively (Table 4). While hypertrophy is a non-adverse adaptive change in mammalian models, it was the earliest histopathologic alteration attributed to MC-LR ingestion in the liver.

Microscopically determined changes in GJIC across MC-LR treatments and sexes were not statistically significant. GJIC has been shown to play a role in toxicity of agents and has been previously shown to decrease in hepatocytes exposed to non-genotoxic compounds [32,35,40]. We previously hypothesized that MC-LR would decrease GJIC. This predicted outcome is driven by the ability of MC-LR to covalently bind to Protein Phosphatase 1/2A. PP1/2A isoforms are found within the nucleus, nucleolus, and in the cytoplast [41]. The covalent binding of MC-LR to PP1/2A, especially within the nucleolus and nucleus, may make it unavailable for transport via GJIC [41]. However, oxidative stress, one of the main causes of cellular damage associated with MC-LR toxicity, has been shown to reduce GJIC in murine hepatocytes in vitro [42]. Results in the current studies showed no significant changes in GJIC amongst exposed mice (Figure 3), suggesting that GJIC does not play an obvious role in increasing hepatotoxicity of MC-LR in this model system. This relationship between MC-LR and GJIC should be explored further, using larger sample groups and dose-response variations.

The difference in MC-LR related mortality and toxicity across sexes in CD-1 mice is perhaps the most intriguing finding of these studies. Histopathologic examination noted a higher incidence of hepatocellular degeneration and necrosis in male mice compared to female mice (Table 4), while clinical chemistry parameter changes were higher in exposed females than exposed males (Table 3). Cellular damage was localized to central veins and midlobular areas, consistent with MC-LR uptake by hepatocytes early in the process of MC-LR transfer into the liver. Upon uptake by the hepatocyte, MC-LR is metabolized or bound to cellular components, which may localize its damage to the hepatocytes immediately outside of central veins [19,43]. This mechanism may also explain the limited and non-significant change rates of GJIC in exposed animals.

Exposure to MC-LR in males produced dose-dependent increases in ALT, AST, ALP, cholesterol, and bilirubin, contrasted by dose-dependent decreases in serum glucose (Figure 1; Table 1). ALT, AST, ALP, cholesterol, and bilirubin levels also increased, while glucose decreased in female mice (Figure 2; Table 2). However, female effects were not dose-dependent and increases occurred at a higher magnitude compared to male mice. Variations in individual chemistry are likely responsible for the lack of a dose-response relationship in females. The observed changes in clinical chemistry parameters indicate the localization of MC-LR toxicity to the liver, with AST/ALP ratios lower than those related to liver cell apoptosis due to non-alcoholic liver disease [21].

Varying oral MC-LR toxicity across sexes of CD-1 mice can be partially attributed to difference in activity of GST levels in male and female CD-1 mice. As MC-LR detoxification is mainly catalyzed by GST enzymes and relies on the production of GSH conjugates for the elimination of MC-LR metabolites, differences in GST activity and GSH concentration can impact toxicity [19]. Previous studies have shown that CD-1 female mice show a lower constitutive GST activity and, therefore, also produce lower levels of GSH conjugates [23,44,45]. To our knowledge, this is the first study to indicate that CD-1 female mice experience more negative liver health outcomes and are more likely to experience MC-LR related hepatotoxicity than male CD-1 mice.

Although human ingestion of MC-LR at such high concentrations is unlikely to occur in nature, animals continue to be at risk of toxicity. Several past events have shown mortalities in dogs and cattle consuming large quantities of cyanotoxin contaminated water [46,47]. These events can often occur in areas heavy in recreational use, small lakes, and agricultural ponds experiencing harmful algal blooms. Furthermore, while this study is not replicative of human ingestion, it lends insight into the toxicity of MC-LR in humans. While such a scenario is rare, cases of human mortality in individuals receiving dialysis treatment with MC contaminated water would be most analogous to MC-LR exposures demonstrated in this study [5].

In conclusion, this study successfully describes the impacts of acute MC-LR exposure on hepatocyte histopathology, clinical chemistry, and GJIC in vivo. This is the first study to find variations in MC-LR toxicity following oral ingestion across sexes in CD-1 mice. Future works will explore potential biomarkers of liver damage, the impact of MC-LR exposure on hepatocellular gene expression and study the adaptive response of exposed hepatocytes. Prospective studies should take into consideration possible differences in MC-LR toxicity between male and female, and further investigate the relationship between MC-LR exposure and GJIC.

## 4. Materials and Methods

### 4.1. Chemical Compounds

MC-LR was purchased from Beagle Bioproducts Inc. (Columbus, OH, USA). The compound was stored in amber glass vials at −20 °C. Aliquots were resuspended in deionized water before being used for animal exposure.

### 4.2. Animals

The preliminary stage of the study (Phase A) used 6 male and 6 female CD-1 mice (Charles River Laboratories; Raleigh, NC, USA). The animals were 8 weeks of age with body weights ranging from 23.3 to 32.9 grams at dosing initiation.

The complete study (Phase B) utilized 30 male and 30 female CD-1 mice from Charles River Laboratories. Animals were 7 weeks of age and weighed 21.6 to 31.5 grams at dosing.

A stratified randomization scheme was used to assign animals to dosing groups with a goal of achieving similar group mean body weights. Males and females were randomized separately for both phases.

### 4.3. Animal Husbandry

All animal husbandry, handling, treatment administration and mouse sample collection was conducted by professional staff at Charles River Laboratories (Spencerville, OH, USA). The Institutional Animal Care and Use Committee of Charles River Laboratories reviewed the protocol and all relevant amendments, issuing initial approval on 25 May 2016 (IACUC No. 20097701), with approval of amendments on 4 January 2017, and 6 January2017 (20097701-MAUP-1). Individual animals, identified using metal ear tags, were housed in wire mesh floor cages and were allowed 6 days of acclimation. Cages were housed in rooms on a 12 h light/dark cycle with temperatures between 21–23°C and a relative humidity of 47–58%. Mice received PMI Nutrition International Certified Rodent Chow No. 5CR4 (14% protein) and municipal water (treated by reverse osmosis and ultraviolet irradiation) *ad libitum* via an automatic watering valve.

### 4.4. Experimental Protocol

Dosing regimens for experimental phases were determined based on LD_50_ values from published literature. A review of past toxicity studies revealed a range of LD_50_ values (3000–10,000 µg/kg/day) based on oral gavage and intraperitoneal (i.p.) injection toxicities across several mouse models [1,14,17,39]. In order to normalize i.p. injection to oral gavage toxicities, i.p. injection toxicities were reduced by a factor of 10, based on previously published data [14]. Due to variations in LD_50_ values, and unknown effect of MC-LR in CD-1 mice, the experiment was divided into two phases: Phase A, an exploratory study to ensure excess toxicity was avoided; Phase B, the complete study using various MC-LR doses.

For Phase A, animals were randomly assigned to three groups and administered 2000 µg/kg/day of MC-LR via oral gavage (Table 5). Group 1 received one treatment daily from days 1 through 7. Group 2 treatments were administered once daily from days 4 through 7. Group 3 treatments were administered once on Day 7.

For Phase B, animals were randomly assigned to three groups and administered concentrations of MC-LR varying by assigned group (Table 6). Treatment was administered once daily from days 1 to 7.

### 4.5. Animal Observations

General health, mortality, and moribundity checks were conducted twice daily throughout the study. A further, once daily cage-side observation was conducted throughout the dosing period (1 to 3 h post-dose) without animal removal. A detailed clinical observation was conducted on the first day of randomization, the first day of dosing, and day 8 for each phase and group. During the clinical observations body weights were also recorded.

### 4.6. Euthanasia and Necropsy

All animals were euthanized by isoflurane inhalation followed by exsanguination. Animals were euthanized rotating across dose groups such that similar numbers of animals from each group, including controls, were necropsied through the day. Animals were subjected to a complete necropsy examination which evaluated musculoskeletal system, all external orifices and surfaces; the cranial cavity and external surfaces of the brain; and the thoracic, abdominal, and pelvic cavities with their associated organs and tissues.

### 4.7. Unexpected Deaths

A necropsy was conducted for animals that died on study during Phase B. Animals were refrigerated prior to necropsy to minimize autolysis. Following death, animals were necropsied and specified tissues were collected and stored. One group 3 female was also euthanized prior to study conclusion, during Phase B due to deteriorating health. Clinical chemistry parameters and tissue samples were retained according to previously planned protocol.

### 4.8. Clinical Chemistry Parameters

Blood samples were collected from all animals (Phase A and B) on Day 8 via a vena cava blood draw under isoflurane anesthesia at gross necropsy. Samples were also collected for animals that were euthanized moribund. Blood samples were processed to serum and evaluated for several clinical parameters. Liver biomarker enzymes and other biochemical parameters were analyzed enzymatically on a Beckman Olympus AU 640e Clinical Chemistry Analyzer (Table 7). A reagent status check was performed daily prior to any analysis. This checks both reagent compartments in the Olympus 640e instrument, and the reagent check must be performed before analysis can continue. Quality control checks were conducted daily to check the accuracy and precision of the analyzer. A minimum of two levels of quality control were run at the beginning and end of each sample run. Any repeat results were reviewed and compared with the original result to determine the results verify each other or a third repeat is required. All the quality control checks and calibrations were conducted by following the Olympus Reagent Guide Clinical Chemistry (Version 2.0. December 2006. Olympus Diagnostic Systems, Olympus America, Inc., Center Valley, PA, USA).

### 4.9. Tissue Collection, Preservation, and Histopathology

In Phase B, livers were grossly examined, weighed, and sectioned for histopathology, fluorescent dye cut-loading, and gene expression analysis. Liver tissues for histopathology were trimmed, embedded in paraffin, sectioned, and mounted on glass slides. Mounted samples were stained with hematoxylin and eosin and histologically evaluated by a board-certified veterinary pathologist.

### 4.10. Florescent Dye Cut-Loading

Immediately following removal and weighing, a portion of the right lobe of each liver was rinsed in 1× PBS, submerged in a solution of 0.04% Lucifer yellow CH in PBS (Millipore Sigma; St. Louis, MO, USA) and cut with a scalpel blade. Cut liver pieces were immersed in dye for 5 min to permit uptake and transfer of dye. Liver samples were rinsed in 1× PBS and fixed in 10% neutral-buffered formalin. After a 4-day fixation, tissues were clarified by soaking in DMSO for 48 h, and embedded in paraffin. Tissues were sectioned transversely to the dye-loaded, cut surface (5 mm) by standard methods.

**Figure 4:**
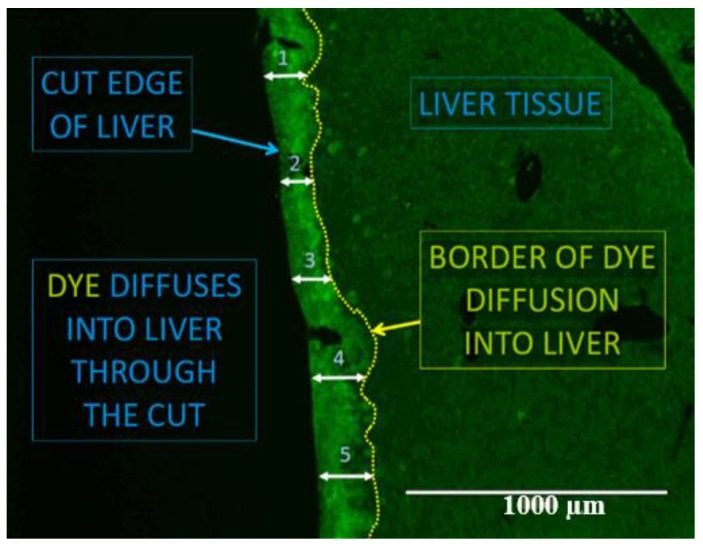
Fluorescent dye cut-loading representative procedure.

Fluorescence microscopy was utilized to evaluate dye perfusion into cut-loaded tissue. Dye-coupled cells were counted at six random points on each fixed liver sample (Figure 4). A representative mean was determined for each liver sample.

### 4.11. Statistical Analysis

For Phase A, data were presented as values individual to each animal. For Phase B, all statistical tests were conducted at the *a* = 0.05 significance level, while pairwise comparisons used 2-sided tests reporting at *a* values of 0.001, 0.01, and 0.05.

Levene’s test was used to assess homogeneity of group variances. Comparisons across these groups were done using ANOVA *F*-test or Kruskal-Wallis test, depending on normality. If ANOVA or Kruskal-Wallis tests were found to be significant, pairwise comparisons were conducted using Dunnett’s or Dunn’s test, respectively. Datasets with two groups were compared using *t*-test or Wilcoxon Rank-Sum test, depending on normality determined by Levene’s test. Differences in cellular gap junction communication across treatments and sexes were examined using ANOVA.

## Figures and Tables

**Table 1:** Male clinical chemistry parameters.

Clinical Parameter	Dose (µg/kg/day)		
	0	3000	4000/5000
AST (U/L)	49.00 ± 2.15	91.67 ± 8.77 ^a^	203.00 ± 80.17 ^c^
ALT (U/L)	39.10 ± 3.34	137.56 ± 22.38 ^b^	419.00 ± 219.08 ^c^
ALP (U/L)	77.20 ± 6.01	116.56 ± 8.03	223.67 ± 25.78 ^c^
TBIL (mg/dL)	0.15 ± 0.01	0.209 ± 0.01	0.60 ± 0.30 ^c^
CHOL (mg/dL)	157.00 ± 8.3	205.11 ± 12.37 ^b^	166.22 ± 11.03
GLUC (mg/dL)	199.90 ± 9.23	159.44 ± 8.33 ^b^	140.89 ± 9.13 ^c^

Significantly different from 0 (µg/kg/day) value: ^a^ = *p* ≤ 0.05; ^b^ = *p* ≤ 0.01; ^c^ = *p* ≤ 0.001 (Dunn). All values reported as mean ± standard error.

**Table 2:** Female clinical chemistry parameters.

Clinical Parameter	Dose (µg/kg/day)		
	0	3000	4000/5000
AST (U/L)	73.50 ± 11.25	952.40 ± 553.54 ^b^	891.43 ± 793.15
ALT (U/L)	63.50 ± 17.43	1574.50 ± 946.57 ^b^	857.00 ± 731.71 ^a^
ALP (U/L)	103.70 ± 5.21	226.70 ± 29.25 ^c^	184.57 ± 17.39 ^a^
TBIL (mg/dL)	0.14 ± 0.01	0.33 ± 0.12	0.23 ± 0.01
CHOL (mg/dL)	109.50 ± 2.99	151.70 ± 10.23	163.86 ± 12.97 ^b^
GLUC (mg/dL)	206.40 ± 8.62	136.78 ± 14.96 ^d^	172.14 ± 24.44

Significantly different from 0 (µg/kg/day) value: ^a^ = *p* ≤ 0.05; ^b^ = *p* ≤ 0.01; ^c^ = *p* ≤ 0.001 (Dunn). Significantly different from group 1 value: ^d^ = *p* ≤ 0.01 (Dunnett).

**Table 3:** Maximal Magnitude of Change in Clinical Chemistry Parameters.

Marker	Abbrev.	Maximal Fold Change Observed	
		Males	Females
Aspartate aminotransferase	AST	4.1	13
Alanine aminotransferase	ALT	10.7	24.8
Alkaline Phosphatase	ALP	2.9	2.2
Cholesterol	CHOL	n/a	1.5
Bilirubin	TBIL	4.1	2.3
Mean Glucose	GLUC	(−0.8)	(−0.7)

**Table 4:** Pathological findings in mice exposed to varying MC concentrations.

Parameters	Males			Females		
Dose (µg/kg/day)	0	3000	5000/4000	0	3000	5000/4000
*N*	10	10	10	10	10	10
Hypertrophy	(0) ^a^	(8)	(10)	(0)	(2)	(9)
Minimal	-	6	1	-	2	3
Mild	-	2	9	-	0	6
Degeneration	(0)	(5)	(10)	(0)	(4)	(7)
Minimal	-	4	2	-	2	0
Mild	-	1	8	-	2	7
Necrosis	(0)	(6)	(7)	(0)	(3)	(2)
Minimal	-	5	2	-	1	1
Mild	-	0	3	-	0	0
Moderate	-	1	2	-	2	0
Hemorrhage	(0)	(1)	(2)	(0)	(0)	(3)
Minimal	-	-	0	-	-	-
Mild	-	1	2	-	-	3

^a^ Parentheses represent total number of animals with the finding.

**Table 5:** Experimental Design for Phase A.

Group	MC-LR Dose (µg/kg/day)	Dose Volume (mL/kg)	Dose Concentration (µg/mL)	Number of Animals	
				Males	Females
1 ^a^	2000	10	200	2	2
2 ^b^	2000	10	200	2	2
3 ^c^	2000	10	200	2	2

^a^ Group 1 was dosed once daily on Days 1–7; ^b^ Group 2 was dosed once daily on Days 4–7; ^c^ Group 3 was dosed once on Day 7.

**Table 6:** Experimental Design for Phase B.

Group	MC-LR Dose (µg/kg/day)	Dose Volume (mL/kg)	Dose Concentration (µg/mL)	Number of Animals	
				Males	Females
1	0 ^a^	10	0	10	10
2	3000	10	300	10	10
3	5000/4000 ^b^	5000/4000 ^b^	500	10	10

^a^ Reverse osmosis deionized (RODI) water; ^b^ Due to mortality observed on Day 1, the dose level was changed to 4000 µg/kg/day on Day 2.

**Table 7:** Clinical Chemistry Parameters Examined During Study.

Alanine aminotransferase ^a^ Aspartate aminotransferase ^a^ Alkaline phosphatase ^a^ Gamma-glutamyltransferase ^a^ Creatine Kinase ^a^ Total bilirubin Urea nitrogen Creatinine Calcium Phosphorus Total protein	Albumin Globulin (calculated) Albumin/globulin ratio Glucose Cholesterol Triglycerides Sodium Potassium Chloride Sample Quality

^a^ Priority for collection.
